# Identification of YABBY Transcription Factors and Their Function in ABA and Salinity Response in *Nelumbo nucifera*

**DOI:** 10.3390/plants12020380

**Published:** 2023-01-13

**Authors:** Shuping Zhao, Yao Zhang, Mengying Tan, Jiao Jiao, Chuyan Zhang, Peng Wu, Kai Feng, Liangjun Li

**Affiliations:** 1 College of Horticulture and Landscape Architecture, Yangzhou University, Yangzhou 225009, China; 2 Joint International Research Laboratory of Agriculture and Agri-Product Safety of Ministry of Education of China, Yangzhou University, Yangzhou 225009, China

**Keywords:** YABBY, salt stress, ABA, transcription factor, *Nelumbo nucifera*

## Abstract

The plant-specific transcription factor family YABBY plays important roles in plant responses to biotic and abiotic stresses. Although the function of YABBY has been identified in many species, systematic analysis in lotus (*Nelumbo nucifera*) is still relatively lacking. The present study aimed to characterize all of the *YABBY* genes in lotus and obtain better insights into *NnYABBYs* in response to salt stress by depending on ABA signaling. Here, we identified nine *YABBY* genes by searching the whole lotus genome based on the conserved YABBY domain. Further analysis showed that these members were distributed on six different chromosomes and named from *YABBY1* to *YABBY9*, which were divided into five subgroups, including YAB1, YAB2, YAB5, INO, and CRC. The analysis of *cis*-elements in promotors revealed that *NnYABBYs* could be involved in plant hormone signaling and plant responses to abiotic stresses. Quantitative real-time PCR (qRT-PCR) showed that *NnYABBYs* could be up-regulated or down-regulated by ABA, fluridone, and salt treatment. Subcellular localization indicated that NnYABBY4, NnYABBY5, and NnYABBY6 were mainly localized in the cell membrane and cytoplasm. In addition, the intrinsic *trans*-activity of NnYABBY was tested by a Y2H assay, which revealed that NnYABBY4, NnYABBY5, and NnYABBY6 are deprived of such a property. This study provided a theoretical basis and reference for the functional research of YABBY for the molecular breeding of lotus.

## 1. Introduction

*Nelumbo nucifera* Gaertn. is a perennial aquatic herbaceous plant that is the main aquatic vegetable in China and has horticultural, ornamental, medicinal, and food values [1,2,3,4,5]. The cultivated areas of lotus have reached more than 600,000 hectares, and lotus cultivation forms a unique high-quality production area with unique freshwater lake natural resources in China [6]. Its products, as a tonic food welcomed by consumers, are rich in starch, protein, a variety of vitamins, and mineral nutrition, with good nutrition and health functions. ‘*Shennong Materia Medica*’, a Chinese pharmacopeia book, listed lotus as a top-quality tonic that could nourish the blood, muscle, and spleen. *Nelumbo nucifera*, as a sessile plant that grows in a complex and changing environment, has evolved a sophisticated defense mechanism to cope with stresses, such as abiotic and biotic stresses [7]. The transcription factor families, such as NAC, bZIP, MYB, YABBY, NF-YB, and AP2, are important regulatory elements for plants to respond to external environmental stresses [8,9].

Among the above transcription factors, YABBY, as a small family, receives a lot of attention in several aspects of plant research [10,11,12]. The structural domains of YABBY members, N-terminal C2C2 domain mediating protein interactions, and C-terminal YABBY domain mediating protein–DNA interactions, are highly conserved in different species. The proline-rich region and the HMG box-like region in the C-terminal are indispensable for DNA binding by YABBY proteins [13]. Six members of YABBY exist in *Arabidopsis*, which can be divided into five major groups, namely YAB2, YAB5, YAB1/ FILAMENTOUS FLOWER (FIL), INNER NO OUTER (INO), and CRABS CLAW (CRC) [14]. In addition, a total of 12, 17, and 7 *YABBY* genes have been identified in cotton, soybean, and grapevine, respectively [11,15,16].

*YABBY* genes participate in many physiological processes of plants, especially in the patterns of leaf differentiation and growth of floral organs [17,18,19]. FIL, YAB2, and YAB5 are expressed in the abaxial region of the above-ground lateral organ primordia and can determine the growth trend of the abaxial side of the cell and promote laminar growth [20,21]. The YAB2 gene is involved in flower morphology and the evolution of laminar styles [22]. CRC and INO mainly play roles in the development of reproductive organs: CRC is necessary for the development of the carpel and promotes the longitudinal growth of the developing gynoecium [23,24,25]; INO is especially expressed only in the outer integument on the abaxial side of the ovule primordium and is suggested to be involved in polar determination [19,26,27]. YAB5 is mainly expressed in the abaxial region of the lateral organs, especially in the midvein region and petiole, where it is most strongly expressed [27]. SWP73B, an important gene for maintaining floral organ characteristics, is also able to regulate leaf development by binding to the promoter region of YAB2/3/5 [28].

Furthermore, YAB genes are also involved in the physiological and biochemical responses of plants to abiotic stresses. Three *YABBY* genes are positively induced by cold stress in paper mulberry [29]. Similarly, the upland cotton *YABBY* genes, *GhYABBY3_At/Dt* and *GhYABBY8_At/Dt*, are also up-regulated by cold stress [15]. *AcYABBY4* increases the salinity tolerance of transgenic *Arabidopsis* lines [12]. *Cis*-element analysis indicated that apple *YABBY* genes may be involved in plant responses to stresses [30]. Furthermore, *GmYABBY10* and *AcYABBY4* have been shown to negatively regulate the transgenic *Arabidopsis* response to drought and salt stresses [11,12]. These studies showed that *YABBY* genes are important regulators of plant resistance to abiotic stresses.

Many articles on abiotic stress have been published, and many transcription factors involved in the responses to abiotic stress in plants have been revealed. Proline-rich extensin-like receptor kinases (PERKs) are involved in the responses to biological and abiotic stresses in wheat [31]. When plants are under abiotic stress, long non-coding RNAs can interact with miRNAs to regulate transcription factors related to the stress response [32]. Many cis-regulatory elements related to abiotic stress have been found in the promoter sequences of ACO genes, indicating that ACO genes play an important role in plants’ responses to abiotic stress [33]. The KCS gene family shows a response to drought stress in barley [34].

*Nelumbo nucifera* Gaertn. growing in the aquatic environment is vulnerable to environmental stimulation, such as salt stress. Seeking salt-stress-related genes is important work in the modern breeding of *Nelumbo nucifera* Gaertn. Although a number of articles on YABBY in other species have been published, there have been few reported on *Nelumbo nucifera* [11,35,36]. In our study, we identified nine YABBY members in lotus. We conducted a systematic bioinformatic analysis of these members. We investigated the relative expression levels of *YABBY* under different treatments containing ABA and high salt. We found that some YABBY family members are sensitive to ABA and salt, which provides a scientific reference for studying the resistance role of YABBY family members in the face of abiotic stresses in plants. Our study provides an important theoretical basis for exploring the molecular mechanisms and regulatory pathways of *YABBY* genes in response to abiotic stresses.

## 2. Results

### 2.1. Identification of NnYABBY Gene Family in Lotus

The suspected YABBY protein sequences of lotus and Arabidopsis were searched for and downloaded from the Nelumbo Genome Database and TAIR website (https://www.arabidopsis.org/, accessed on 10 September 2022). Both protein sequences were inserted into the online tool pfam to confirm the conservative domain of YABBY. Using the conservative domain as a query, nine *YABBY* gene family members were identified from the lotus genome. Nine *NnYABBY* genes were named from *NnYABBY1* to *NnYABBY9* according to the order of their chromosomal locations. The lengths of nine *NnYABBY* gene-encoded proteins varied from 146 to 211 amino acids (Table 1). Each NnYABBY family member has a different protein molecular weight. The smallest protein is *NnYABBY5*, and it has a weight of 16481.96 kDa. The longest protein is *NnYABBY9*, and it has a weight of 23365.56 kDa. The NnYABBY protein sequences had large variations in the theoretical pI (ranging from 5.20 to 9.61). For nine NnYABBY proteins, it was predicted that all proteins did not contain signal peptides.

### 2.2. Phylogenetic Analysis

**Figure 1:**
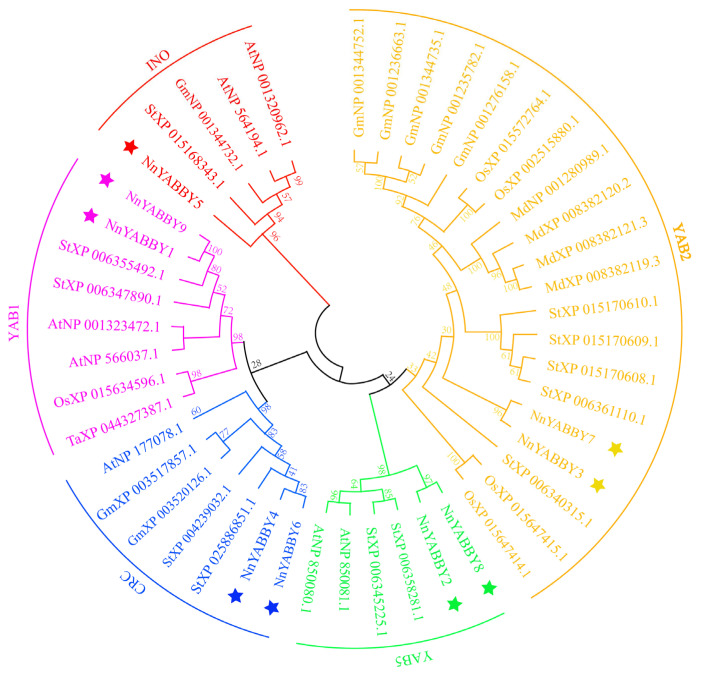
Phylogenetic tree of interspecific *YABBY* relationships involving lotus, potato, soybean, rice, wheat, and *Arabidopsis*.

We selected species such as potato, rice, wheat, soybean, and Arabidopsis for phylogenetic tree analysis, along with lotus (Figure 1). According to previous studies, YABBY was classified into five categories, including YAB1, YAB2, YAB5, INO, and CRC. NnYABBY3 and NnYABBY7 belong to the YAB2 group. The YAB5 group includes two members, NnYABBY2 and NnYABBY8. NnYABBY1 and NnYABBY9 belong to the YAB1 group. NnYABBY5 belongs to the INO group. NnYABBY4 and NnYABBY6 belong to the CRC group.

### 2.3. Gene Structures and Motif Analysis of NnYABBYs

**Figure 2:**
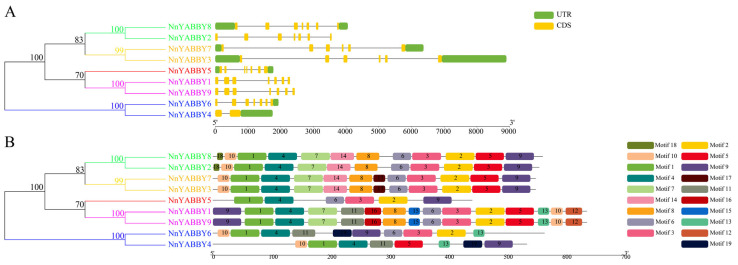
Gene structures and motif analyses of *NnYABBYs*. (**A**) Gene structures of *NnYABBYs*; (**B**) Conserved motif analysis of *NnYABBYs*. Motifs were analyzed by using the MEME website.

We analyzed nine *NnYABBY* gene structures by using the GFF annotation file of lotus (Figure 2). We found that most *NnYABBY* gene members had seven exons. *NnYABBY3* and *NnYABBY7* had six exons. *NnYABBY4* had the fewest exons, only two. *NnYABBY1* and *NnYABBY9* belonged to the same subgroup in the phylogenetic tree, and they had extremely similar gene structures. The situation was the same for other *NnYABBY* gene family members. *NnYABBY* gene family members from the same subfamily had more similar gene structures, while the different subfamily members had more different gene structures.

The prediction of the motif showed that all proteins encoded by *NnYABBY* gene family members had motif 2 near the N terminus, which consisted of the C2C2 zinc-finger domain, which was consistent with the specific structure of the *YABBY* gene family. We also found that the gene members from the same subfamily, such as *NnYABBY1* and *NnYABBY9*, had similar motifs. *NnYABBY1* and *NnYABBY9* were from the YAB1 subfamily, and both had motif 12, motif 15, and motif 16, which can distinguish them from other family members. *NnYABBY4* and *NnYABBY6* were from the CRC subfamily, and both had motif 19, which may be unique to the CRC subfamily. *NnYABBY2* and *NnYABBY8* from the YAB5 group had the specific motif 18 and the YAB2 group, including *NnYABBY3* and *NnYABBY7,* had the specific motif 17. The phylogenetic tree was analyzed with the gene structure and motifs together, and we hypothesized that the gene structure and characteristics of the motifs had an important influence on distinguishing the different subfamilies. We also hypothesized that they had significant impacts on the function of NnYABBY genes from different subfamilies.

### 2.4. Chromosome Localization and Collinearity Analysis

**Figure 3:**
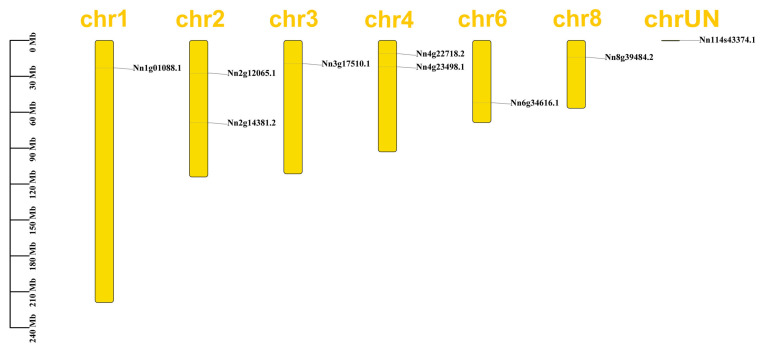
Chromosomal locations of *NnYABBY* genes in lotus. The yellow represents the chromosome and the chromosome number is on top of the bars. There is no correlation between the length of the bars and the size of the chromosomes. The map distance is on the left side of the chromosomes and its unit is megabases (Mb).

We used the GFF file downloaded from the Nelumbo Genome Database to extract the position of each *NnYABBY* family member on the chromosome and visualized it using TBtools (Figure 3). *NnYABBY1* was located on an unknown chromosomal segment. Other *NnYABBYs* were distributed on each chromosome. Chromosome 2 and chromosome 4 contained two *YABBY* gene family members.

**Figure 4:**
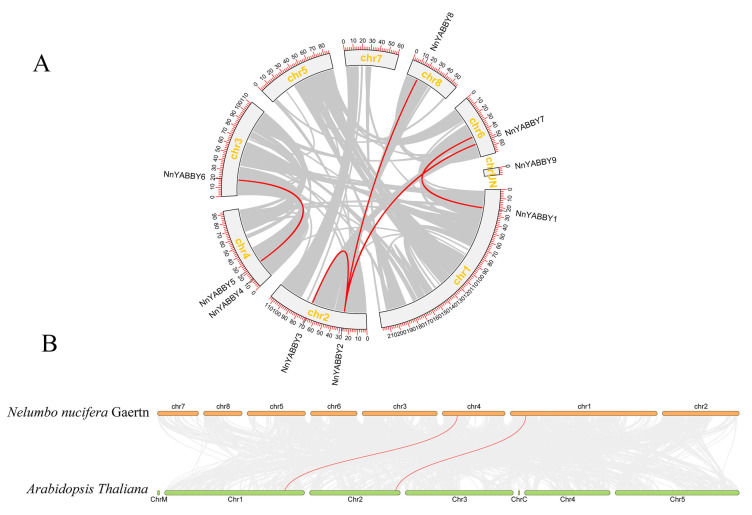
Collinearity analysis of the *NnYABBY* gene family in the lotus. (**A**) Intra-specific collinearity analysis. (**B**) Collinearity analysis between *Nelumbo nucifera* Gaertn and *Arabidopsis thaliana*. Gene pairs are represented by the red lines.

To investigate whether there was tandem replication or fragment replication in the chromosomes of lotus, we performed intra-species covariance analysis [37]. The collinearity analysis was visualized by using MCScanX in TBtools (Figure 4). Five pairs of gene-duplication genes were found in nine *NnYABBY* gene family members, including *NnYABBY1/NnYABBY7*, *NnYABBY2/NnYABBY8*, *NnYABBY2/NnYABBY7*, *NnYABBY2/NnYABBY3*, and *NnYABBY5/NnYABBY6*. Gene duplication occurs more frequently in chromosome 2 than in other chromosomes. There were no fragment duplication gene pairs in chromosome 5 for lotus and *Arabidopsis thaliana*. During the long evolution of lotus, the emergence of gene duplication events facilitated the creation of new genes and the creation of genes’ new functions.

To understand the types of genes and the relative sequences of conservation among different species that diverged from the same ancestral type, we conducted collinearity analysis between lotus and *Arabidopsis thaliana*. Two pairs of gene duplication genes occurred between lotus and *Arabidopsis thaliana*. These gene pairs played an important role in driving the evolution of plants.

### 2.5. Protein Structure Analysis and Protein Interaction Network

**Figure 5:**
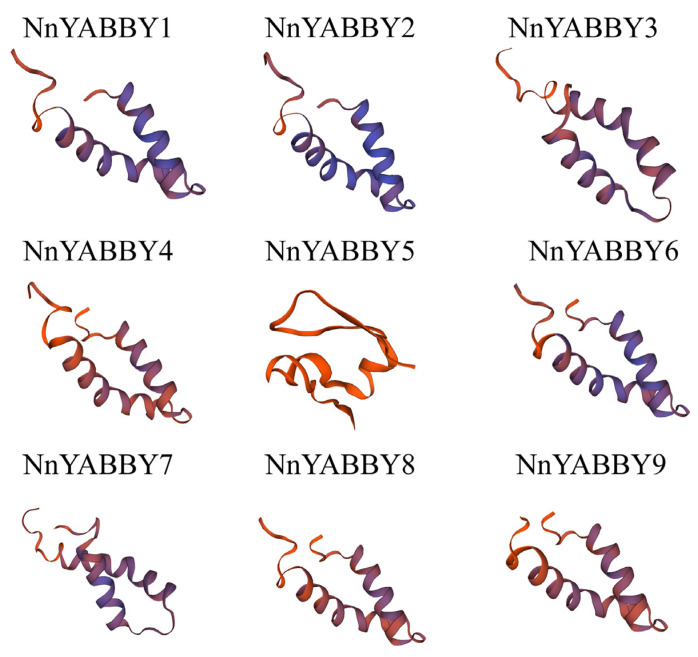
Tertiary structure of NnYABBY proteins. Through the protein sequence query, the default parameter Settings in SWISS are used to map the tertiary structures.

The secondary structures of the NnYABBY protein family in lotus consist of an alpha helix, extended strand, and random coil. Random coils make up the largest part of the secondary structures, followed by extended strands and alpha helices. The tertiary structures of the proteins were mapped by SWISS-MODEL (Figure 5). The structures of gene members from the same subfamily were more similar than those of other members. For example, NnYABBY1/NnYABBY9, NnYABBY2/NnYABBY8, and NnYABBY3/NnYABBY7 were from the same subfamilies and their protein structures were more similar than those of others. The differences in protein structures corresponded to the classification of NnYABBY family members and may contribute to their different functions in lotus roots.

**Figure 6:**
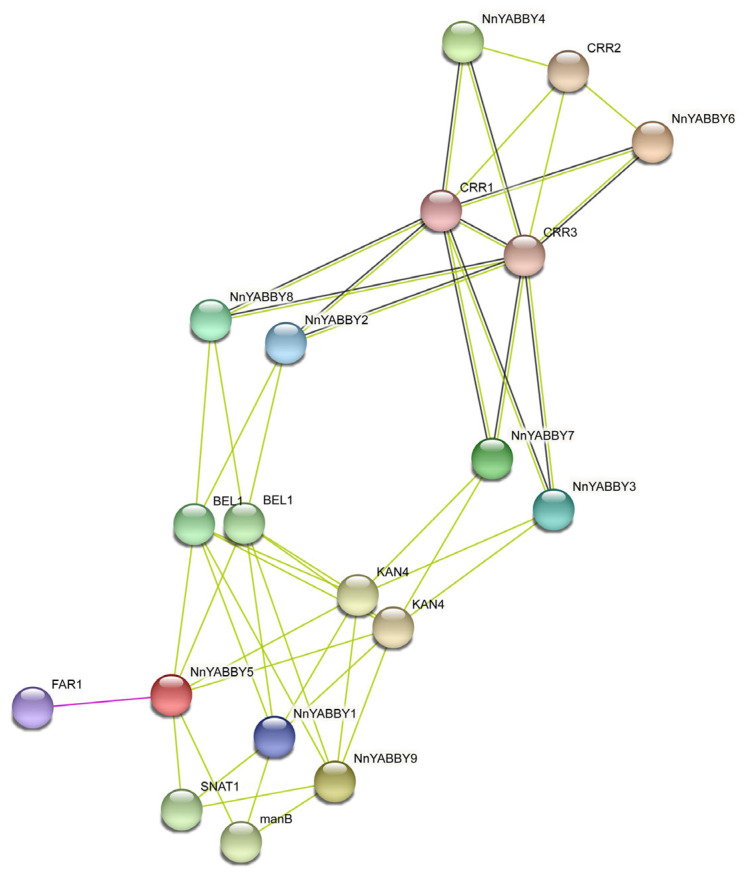
Interaction network of NnYABBY in lotus. The network type was a full STRING network. Different line colors indicate the type of interaction evidence.

We entered nine NnYABBY protein sequences into the STRING website and used the default parameters to visualize the network of NnYABBY protein interactions (Figure 6). Interaction networks of single YAB proteins were also constructed (Figure S1). In the network, nine YAB proteins needed to interact with other proteins to be able to connect to a large network of protein interactions, according to the predictions of the STRING system. Interestingly, NnYABBY proteins from the same subfamily were more similarly positioned on the interaction network and the predicted proteins that interacted with them were also identical. BEL2, KAN4, and CRR could interact with multiple NnYABBY family members. KAN4, a member of the GARP family, is extensively involved in the flavonoid synthesis pathway and is able to regulate the flavonoid content of seeds in Arabidopsis [38]. The protein interactions of NnYABBY with KAN4 suggest that NnYABBY may be involved in flavonoid synthesis and regulates the expression of related genes in the flavonoid synthesis pathway.

### 2.6. Analysis of cis-Acting Elements in Promoter Regions of NnYABBY Genes

**Figure 7:**
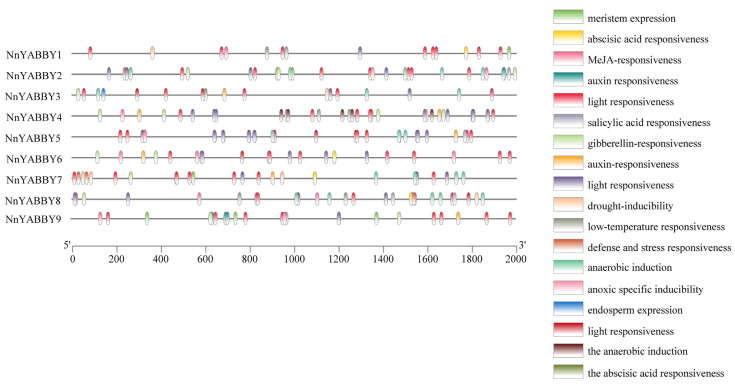
*cis*-acting regulatory elements analysis of *NnYABBY* genes in lotus. The upstream 2000 bp of each NnYABBY member was used as the promoter sequence to query and analyze the *cis*-acting regulatory elements in PlantCARE. Different colors indicate different *cis*-acting regulatory elements.

To understand the response of *NnYABBY* to biotic and abiotic stresses, we analyzed the promoter sequence of *NnYABBY* for *cis*-acting elements (Figure 7). A total of eighteen different *cis*-acting elements were identified in nine *NnYABBYs*. First, a variety of phytohormone response elements were found, including elements for abscisic acid (ACGTG motif), auxin (AACGAC motif), salicylic acid (CCATCTTTTT motif), and gibberellin (TCTGTTG motif). Second, we also found stress-responsive elements in promotors, such as elements for drought (CAACTG motif), low-temperature (CCGAAA motif), anaerobic (AAACCA motif), and anoxic conditions. Third, several light-responsive elements were widely distributed among the nine *NnYABBYs* which were also the most numerous cis-acting elements. Therefore, *NnYABBY* genes may be involved in a variety of physiological and biochemical reactions. Different *NnYABBYs* have different cis-acting elements, so they may have different functions during plant growth and development.

### 2.7. Expression Patterns by Quantitative Real-Time PCR (qRT-PCR)

**Figure 8:**
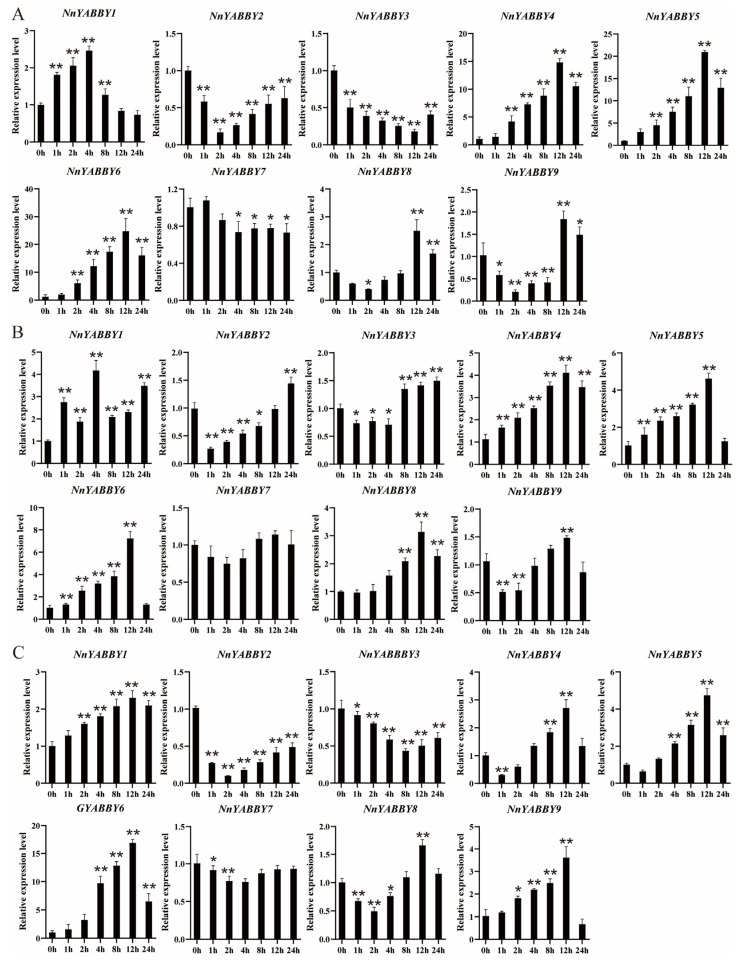
qRT-PCR analysis of *NnYABBY* in lotus leaves under different hormone treatments. (**A**) Expression levels of *NnYABBYs* in lotus leaves under ABA stress. (**B**) Expression levels of *NnYABBYs* in leaves sprayed with ABA and FL. (**C**) Expression levels of *NnYABBYs* in lotus leaves under NaCl stress. (*) represents *p* values of <0.05 and is considered statistically significant. (**) represents *p* values of <0.01 and is considered highly statistically significant.

In order to explore the response of YABBY members to hormone stress, lotus leaves were sprayed with three different hormone treatments, including ABA, ABA+FL, and NaCl. Blades of the same leaf area were selected for qPCR experiments to ensure the accuracy of the results. After spraying the lotus leaves with 100 μmol/L abscisic acid (ABA), all nine *YABBY* members responded to the ABA treatment to varying degrees (Figure 8A). Except for *NnYABBY2*, *NnYABBY3*, and *NnYABBY7*, the expression levels of the other members were up-regulated, with *NnYABBY4*, *NnYABBY5*, and *NnYABBY6* episodes occupying peaks. ABA treatment resulted in the down-regulation of NnYABBY2 and *NnYABBY3* expression after spraying. ABA treatment had no obvious effect on *NnYABBY7,* and its expression did not seem to change. Fluridone, an inhibitor of ABA, was sprayed with ABA together, and the concentration of the FL solution was the same as that of ABA [27]. We hope to prove whether these *NnYABBY* genes are actually regulated by ABA from the other side. We expected that the expression of some genes positively regulated by ABA would return to normal if we sprayed ABA and Fluridone on the lotus leaves at the same time. The results of the experiment (Figure 8B) agreed with our expectation. The expression levels of *NnYABBY4*, *NnYABBY5*, and *NnYABBY6*, which were up-regulated by ABA, became normal when we sprayed ABA and Fluridone. The expression levels of *NnYABBY2* and *NnYABBY3* were down-regulated within eight hours when sprayed with ABA and FL and up-regulated after 8 h when sprayed with ABA and FL. The expression levels of other members were up-regulated after spraying with ABA and FL. Spraying with NaCI could up-regulate the expression levels of *NnYABBY1*, *NnAYBBY6*, and *NnYABBY9*. *NnYABBY4*, *NnYABBY5*, and *NnYABBY6* had the most obvious responses to hormones (Figure 8C). They may play important roles in hormone responses and salt stress responses.

### 2.8. Subcellular Localization

**Figure 9:**
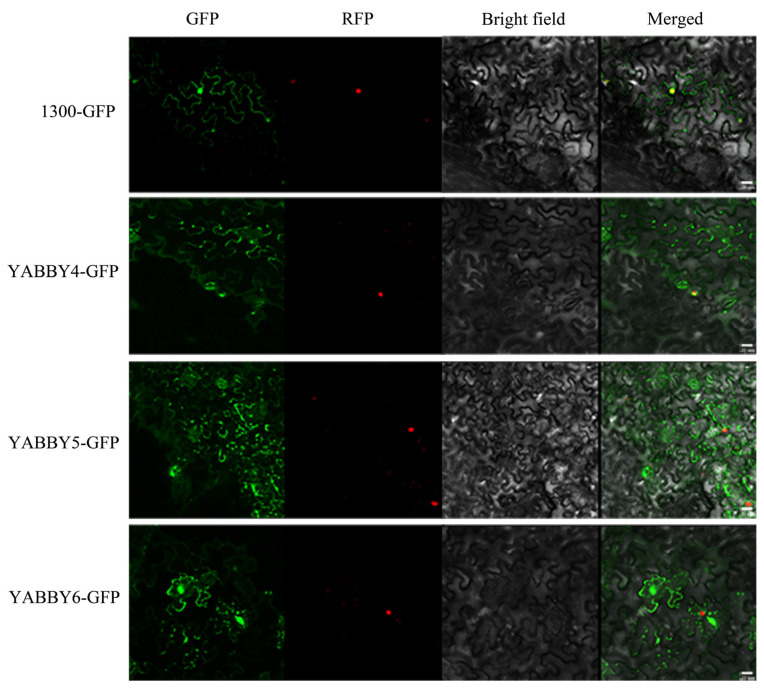
Subcellular localization of the YABBY proteins in lotus. The picture above is the control and that below shows NnYABBY5, NnYABBY6, and NnYABBY7. Scale bar = 20 μm.

According to the quantitative results (Figure 8), the relative expressions of *NnYABBY4*, *NnYABBY5*, and *NnYABBY6* changed most significantly under various hormone treatments. Subcellular localization can reflect the specific locations of the proteins encoded by YABBYs. To better understand the molecular role of YABBY in response to hormones and stress in lotus leaves, the above three members were selected for subcellular location. The control protein we used was 1300-GFP, and its green fluorescence appeared in the nucleus, cytoplasm, and cell membrane. The green fluorescence of NnYABBY4, NnYABBY5, and NnYABBY6 proteins all appeared on the cell membrane and cytoplasm. The cell membrane and cytoplasm are probably the most important places where NnYABBY4, NnYABBY5, and NnYABBY6 perform their functions (Figure 9).

### 2.9. Determination of Transcriptional Activation Function

**Figure 10:**
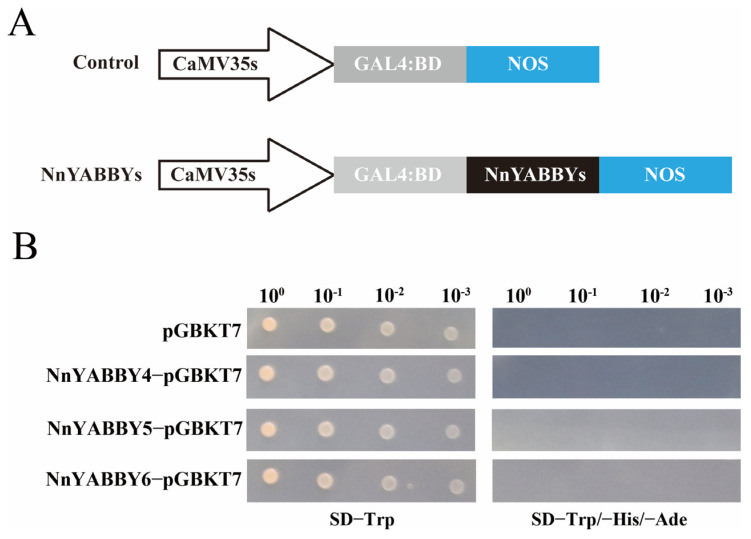
Transcription activation analysis of the three selected NnYABBY proteins. (**A**) Schematic representation of the construction of the fusion plasmid. (**B**) Transcription activation analysis of the 3 selected NnYABBY proteins. pGBKT7 was used as the control.

The full-length cDNA of *NnYABBY4*, *NnYABBY5,* and *NnYABBY6* was cloned into the PGBKT7 vector, and its self-activation ability was verified by a yeast experiment (Figure 10). The negative control pGBKT7, NnYABBY4, NnYABBY5, and NnYABBY6 could grow on SD/−Trp medium, which confirmed that the experimental procedures and practices were correct. Their inability to grow monoclonal colonies on SD/−Trp/−Ade/−His medium indicated that NnYABBY4, NnYABBY5, and NnYABBY6 lacked self-activation ability. They may require the help of other proteins to function and could be used as bait to search for genes that interact with them in cDNA libraries to further analyze the potential functions of NnYABBY proteins.

## 3. Discussion

The *YABBY* family has a typical zinc-finger structural domain and a YABBY structural domain, which are specific transcription factors. In addition to maintaining organ polarity and meristem activity, YABBY proteins can also promote the growth of lamina by regulating the signal transduction processes between the front and back of the leaf [39]. Six members of the *YABBY* gene family in *Arabidopsis* have different expression patterns. The expressions of FIL and YAB3 are located in the abaxial domain of lateral organs [18,21,27]. YAB2 and YAB5 are expressed in abaxial regions of the aboveground lateral organ primordia, whereas the other two (CRC and INO) are expressed in the abaxial domains of the carpels and the outer integument of the ovules, respectively [21,39]. FIL, YAB2, YAB3, and YAB5 have functions in the establishment of leaf polarity, the general genetic program of leaf expansion, and leaf formation [27]. When the genes for FIL, YAB2, and YAB5 were mutated in *Arabidopsis* and thus became non-functional, we found that the mutant leaves lost their abaxial and adaxial identities in *Arabidopsis* [40,41]. The mutants of these *YABBY* genes could bring about radial filamentous floral organs [20,42]. Members of the YABBY family have a role in the outer tepals and leaf development, and when they lose their function, it leads to a reduction in leaf growth [43].

Although articles about the YABBY family have been reported for other species, there are few reports of the YABBY family in lotus. The number of *YABBY* genes in lotus still needs to be determined. Therefore, we identified nine members of the *NnYABBY* gene family in lotus by searching the genome database of lotus based on the raw HMM models for the conserved YABBY domain and C2–C2 domain. Based on the taxonomy of the model plants and numerous well-studied species, we divided NnYABBY in lotus into five subfamilies [44,45]. According to the genome sequence information, the gene structure of *NnYABBY* family members was visualized (Figure 2A). Different *NnYABBY* family members contain different numbers of exons and introns. The MEME website was used to visualize the motif structure of YABBY members (Figure 2B). Members of the *NnYABBY* gene family from the same subfamily had similar motif structures. Gene structure and motif structure are likely to be an important basis for dividing different subfamilies. Different motifs may lead to different functions of YABBY family members, which lead to different roles in plant growth and development. Almost every subfamily has its own unique motif, which is not found in other subfamilies. Motif 18 only appeared in NnYABBY2 and NnYABBY8 from the YAB5 subgroup. The YAB1 subfamily has the most unique motifs, including motif 12, motif 15, and motif 16. NnYABBY3 and NnYABBY7 in the YAB2 subfamily have motif 17. Each unique motif has value to be further investigated, and they may be closely related to the unique functions of their respective subfamilies. Motif 19 is unique to the CRC subfamily, which is required for carpel development. It is likely that motif 19 is an important functional region of the CRC family, which could promote the longitudinal growth of the developing gynoecium, suggesting that motif 19 has the possibility to be related to a unique function in the carpel development of lotus. Nine *NnYABBY* gene family members were evenly distributed on the eight chromosomes of lotus (Figure 3). Collinearity analysis showed that there were five pairs of gene duplication in the eight chromosomes of lotus (Figure 4). Between the two species of lotus and *Arabidopsis*, two pairs of gene duplication occurred. The condition of gene pair duplication is possibly related to the division of *NnYABBY* family members. The secondary protein structures of the YABBY family members were predicted using the Phyre2 website, and the tertiary structures were visualized using the SWISS website. Each *NnYABBY* family member had multiple genes that interacted with it and were involved in multiple plant growth and development processes together. Valuable genes interacting with YABBY remain to be discovered. The prediction of protein interaction networks for nine *NnYABBY* members showed that KAN4, BEL1, and CRR1/3 could interact with several *NnYABBY* gene family members. CRR, as a dihydrodipicolinate reductase, functions in the lysine biosynthetic pathway and is able to expand the ovaries of rice [46,47]. YABBY proteins are likely to interact with CRR1, and thus participate in the cyclic electron transport around photosystem I. The biological processes in plants that YABBY members are involved in by interacting with other genes remain to be discovered, and this is of great significance. CRR3 was found to be a disease-resistance gene in *Brassica rapa* [48]. Among these stresses, salt stress, as the main environmental stimulus affecting plant growth and development, has been widely investigated in many crops (Shavrukov, 2013 #72; Shavrukov, 2013 #72; Shavrukov, 2013 #72; Shavrukov, 2013 #72; Shavrukov, 2013 #72) [49]. These studies have unearthed and identified many excellent plant salt-tolerance genes (Bian, 2020 #73; Gong, 2020 #74) [50,51]. Among our quantitative results (Figure 8), YABBY4, 5, and 6 showed high changes in relative expression under conditions of hormonal stress and salt stress. Therefore, we hypothesize that YAB is likely to be involved in the responses to biotic and abiotic stresses through binding to certain genes. The results of self-activation verification experiments showed that YABBY4, YABBY5, and YABBY6 had no self-activation activity. They probably need some interacting proteins to bind to them in order to function in lotus. It is necessary to discover the protein that could bind to YABBY members.

Lotus is a persistent perennial herb in the lotus family and is one of the most important aquatic vegetables in China [52]. This aquatic vegetable is rich in nutrients, mainly starch, fat, sugar, protein, alkaloid, riboflavin, flavonoids, carotene, vitamin C, and so on [53,54]. Lotus root has not only edible value and medicinal value, but is also an important commodity for foreign exchange. However, salt stress has an important impact on the yield and quality of lotus root [55]. Therefore, determining the key resistance genes is an important task to breed high-quality and high-yielding lotus root varieties. Our results suggest that YABBY4, YABBY5, and YABBY6 may be key genes for improving stress tolerance in lotus roots, which is likely to improve the lotus root yield under biotic and abiotic stresses.

## 4. Materials and Methods

### 4.1. Identification of YABBY Family Members in Lotus

Genome data, cDNA sequences, and peptide sequences of lotus (*Nelumbo nucifera* Gaertn.) were downloaded from the Nelumbo Genome Database (http://nelumbo.biocloud.net/nelumbo/home, accessed on 17 December 2022) [2]. We identified the conserved domain of YABBY by analyzing the *Arabidopsis* YABBY sequences obtained from the TAIR10 database (https://www.arabidopsis.org/, accessed on 17 December 2022) using the pfam website (http://pfam.xfam.org/search/sequence, accessed on 17 December 2022). Then, we identified all members of the YABBY family in lotus by tBLASTN. These YABBYs’ sequence information was used to predict the physicochemical properties of proteins, such as the amino acid length, theoretical isoelectric point, and signal peptides.

### 4.2. Phylogenetic Analysis

The YABBY protein sequence information of wheat, potato, maize, and rice was downloaded from the NCBI database (https://www.ncbi.nlm.nih.gov/, accessed on 17 December 2022). Protein sequences were used for phylogenetic analysis. The MEGA11 software was used to construct a neighbor-joining phylogenetic tree for YABBY family members [4,56]. The pair-wise estimates of the genetic distances were used to calculate the branch lengths in a bootstrap analysis of 1000 replicates.

### 4.3. Gene Structure Analysis and Motif Identification

The exon–intron structures of the NnYABBY family genes were analyzed based on the GFF annotation file obtained from the Nelumbo Genome Database. Motifs of the NnYABBY proteins were identified using MEME’s online tools (http://meme-suite.org/tools/meme, accessed on 17 December 2022) [57]. The number of motifs was set to 10. The structure view was drawn by TBtools [58].

### 4.4. Analysis of NnYABBY Protein Structure and the Network of Proteins Interaction

The main forms of the secondary structures of NnYABBY proteins include α-helices, β-folds, and random coils. We predicted the secondary structure of NnYABBY proteins using the Phyre2 website (http://www.sbg.bio.ic.ac.uk/~phyre2/html/page.cgi?id=index, accessed on 17 December 2022). The SWISS website was used to predict the tertiary structures of NnYABBY proteins and export the corresponding model images (https://swissmodel.expasy.org/, accessed on 17 December 2022).

The STRING database (https://cn.string-db.org/, accessed on 17 December 2022) is one of several online resources dedicated to organism-wide protein association networks [59]. The protein interaction network of NnYABBY members was analyzed using the STRING website.

### 4.5. Chromosome Localization and Collinearity Analysis

We used the annotation GFF files of the lotus genome to visualize the chromosomal localization of *NnYABBY* genes using the TBtools software. We predicted collinearity between genomes and visualized it by using MCScanX and Circos in TBtools [60].

### 4.6. Analysis of cis-Acting Elements in Promoter Regions of NnYABBY Genes

The upstream 2000 bp of every NnYABBY gene was used as its promoter sequence. The *cis*-acting elements were analyzed using PlantCARE (http://bioinformatics.psb.ugent.be/webtools/plantcare/html/, accessed on 17 December 2022). TBtools was used to visualize the number of cis-acting elements in promoters.

### 4.7. Expression Patterns by Quantitative Real-Time PCR (qRT-PCR)

All material plants were grown in the aquatic vegetable experimental field at Yangzhou University, Yangzhou, China. Lotus plants that had grown for two weeks were used in the qRT-PCR experiment. We applied different treatments to the lotus seedlings, including ABA, ABA+FL, and NaCl. The concentration of the ABA and FL solutions was 100 μmol/L. The concentration of the NaCl solution was 200 mmol/L. Untreated lotus was used as the control. We collected samples at 0, 2, 4, 8, 12, and 24 h after various treatments, then dropped them into liquid nitrogen immediately and stored them at −80 °C. The total RNA was extracted using a polysaccharide polyphenol plant total RNA extraction kit (Proteinssci Company). We used 1% agarose gel for electrophoresis, and the results showed three clear and bright bands, especially the 28s band, indicating that the quality of RNA extraction was good. Then, the cDNA was prepared by using reverse transcription kits (Vazyme company, Nanjing, China). This kit contained 5× gDNA Wiper Mix, which can eliminate gDNA contamination in RNA samples effectively. Experiments were conducted according to the system requirements of the reverse transcription kit, and the amount of RNA added to each sample was 1 μL. The cDNA of each sample for qPCR was diluted to 300 ng/μL. We designed the specific qRT-PCR primers (Table S1) based on the coding sequences (CDS) of every NnYABBY gene on NCBI. ChamQ Universal SYBR qPCR Master Mix (Vazyme Biotech, Nanjing, China) was used in the qPCR experiments. The mix solution of qRT-PCR was 2 × ChamQ SYBR qPCR Master Mix, 10 μL; Primer F, 1 μL; Primer R, 1 μL; Template cDNA, 1 μL; and ddH_2_O to 20 μL. The qPCR procedure was set up according to the requirements of the kit above. The data were quantitatively analyzed by the 2^−ΔΔCT^ method [61]. The PCR procedure was run using CFX Connect (BIO-RAD, America). NnTUA was used as a reference gene in the qRT-PCR experiments (Zhao, 2022 #80) [6]. The primers for the TUA gene can be viewed in supplemental data (Table S1).

### 4.8. Subcellular Localization

The CDS sequences of NnYABBYs whose stop codons were removed were cloned using 2×Heiff Canace Plus PCR Master Mix (YEASEN, Shanghai, China). NnYABBYs were respectively built into the pCAMBIA-1300 vectors’ fluorescent protein N-terminal GFP using Hieff Clone Plus One Step Cloning Kit (YEASEN, Shanghai, China). Two restriction sites, *Sac*I and *Xba*I, were used to construct the vector. NnYABBYs with GFP fusion expression vectors and empty vectors were transferred into the leaves of tobacco (*Nicotiana benthamiana*). The tobacco plants were kept in the dark for 3 days. Then, we observed them under a confocal laser scanning microscope (TCS SP8 STED, Wetzlar, Germany).

### 4.9. Determination of Transcriptional Activation Function

NnYABBYs CDS was cloned into a pGBKT7 vector and these plasmids were transformed into a Y2H yeast strain. Two restriction sites, *Bam*HI and *Eco*RI, were used to construct the vector. The transformed yeast strain grew on SD/-Trp plates and SD/-Ade/-His/-Trp plates at 30 °C for 3–6 days.

### 4.10. Statistical Analysis

The statistical analysis method we used was Student’s *t*-test in the relevant experiments. *p* values of <0.05 were considered statistically significant (*), and *p* values of <0.01 were considered highly statistically significant (**).

## 5. Conclusions

In conclusion, a total of nine *NnYABBY* gene members of the lotus were identified and systematically analyzed. The results showed that *NnYABBY* genes from the lotus could be classified into five different subgroups, YAB1, YAB2, YAB5, INO, and CRC. The gene duplication phenomenon may contribute to the evolution of lotus and the expansion of *NnYABBY* family genes. The prediction of the protein interaction network revealed that NnYABBYs may be involved in the regulation of flavonoid biosynthesis. The qRT-PCR analysis indicated that *NnYABBY4*, *NnYABBY5,* and *NnYABBY6* are sensitive to ABA and salt treatment. They may be involved in the physiological responses of plants to abiotic stresses. NnYABBYs have tremendous research value in modern genetic and biological techniques.

## Figures and Tables

**Table 1:** Physicochemical properties of NnYABBY proteins in *Nelumbo nucifera* Gaertn.

ID	Name	Number of Animo Acids (aa)	Molecular Weight (Da)	Theoretical pI	Aliphatic Index	Grand Average of Hydropathicity (GRAVY)
Nn1g01088.1	*NnYABBY1*	211	23301.43	7.11	78.53	−0.318
Nn2g12065.1	*NnYABBY2*	184	20836.82	7.60	75.33	−0.364
Nn2g14381.2	*NnYABBY3*	182	20132.84	8.22	75.00	−0.422
Nn3g17510.1	*NnYABBY4*	177	20250.13	5.20	81.92	−0.168
Nn4g22718.2	*NnYABBY5*	146	16481.96	9.11	74.11	−0.484
Nn4g23498.1	*NnYABBY6*	187	21161.98	9.61	62.03	−0.786
Nn6g34616.1	*NnYABBY7*	182	20146.81	7.68	78.24	−0.419
Nn8g39484.2	*NnYABBY8*	186	20948.01	8.82	77.15	−0.351
Nn114s43374.1	*NnYABBY9*	211	23365.56	7.69	77.58	−0.311

## Data Availability

Not applicable.

## Supplementary Materials

The following supporting information can be downloaded at: https://www.mdpi.com/article/10.3390/plants12020380/s1, Figure S1: Diagram of the interaction network between YABBY and other proteins; Table S1: Primers in this study; Table S2: Secondary structure prediction of the NnYABBY protein sequence.
